# Differential coexpression networks in bronchiolitis and emphysema phenotypes reveal heterogeneous mechanisms of chronic obstructive pulmonary disease

**DOI:** 10.1111/jcmm.14585

**Published:** 2019-08-16

**Authors:** Jiangyue Qin, Ting Yang, Ni Zeng, Chun Wan, Lijuan Gao, Xiaoou Li, Lei Chen, Yongchun Shen, Fuqiang Wen

**Affiliations:** ^1^ Division of Pulmonary Diseases, Department of Respiratory and Critical Care Medicine, State Key Laboratory of Biotherapy of China West China Hospital of Sichuan University Chengdu China

**Keywords:** bronchiolitis, chronic obstructive pulmonary disease, differential coexpression, emphysema, phenotype

## Abstract

Chronic obstructive pulmonary disease (COPD) is a heterogeneous disease with multiple molecular mechanisms. To investigate and contrast the molecular processes differing between bronchiolitis and emphysema phenotypes of COPD, we downloaded the GSE69818 microarray data set from the Gene Expression Omnibus (GEO), which based on lung tissues from 38 patients with emphysema and 32 patients with bronchiolitis. Then, weighted gene coexpression network analysis (WGCNA) and differential coexpression (DiffCoEx) analysis were performed, followed by gene ontology (GO) and Kyoto Encyclopedia of Genes and Genomes enrichment analysis (KEGG) analysis. Modules and hub genes for bronchiolitis and emphysema were identified, and we found that genes in modules linked to neutrophil degranulation, Rho protein signal transduction and B cell receptor signalling were coexpressed in emphysema. DiffCoEx analysis showed that four hub genes (IFT88, CCDC103, MMP10 and Bik) were consistently expressed in emphysema patients; these hub genes were enriched, respectively, for functions of cilium assembly and movement, proteolysis and apoptotic mitochondrial changes. In our re‐analysis of GSE69818, gene expression networks in relation to emphysema deepen insights into the molecular mechanism of COPD and also identify some promising therapeutic targets.

## 1 INTRODUCTION

Chronic obstructive pulmonary disease (COPD) affects millions of people all over the world, and it is associated with high morbidity and mortality.^1^ This incurable lung disease, which is characterized by progressive airflow obstruction involving emphysematous destruction of lung parenchyma and mucus hypersecretion with bronchiolitis,^2^ is estimated to become the third most common cause of death by 2030.^3^ The challenge of managing and treating the disease is due in part to a lack of effective biomarkers and disease‐modifying therapies.

The two classical clinical phenotypes of COPD are bronchiolitis and emphysema.^4^ Patients with bronchiolitis display persistent inflammation, goblet cell hyperplasia and mucin hyperexpression in the airway,^5^ increasing intraluminal mucus, wall muscle fibrosis and airway stenosis.^6^ Emphysema involves elastolytic destruction of the alveolar wall without obvious fibrosis and loss of normal lung tissue.^7^ COPD patients with either the bronchiolitis or the emphysema phenotype differ in their clinical characteristics and treatment response. Emphysema patients seem to present worse pulmonary function and greater dyspnoea than bronchiolitis patients.^8^ Long‐acting beta‐agonist and inhaled corticosteroid treatment leads to greater improvement in lung function and dyspnoea in obstruction‐dominant COPD patients than in emphysema‐dominant patients.^9^ This suggests that the bronchiolitis and emphysema phenotypes of COPD occur via different mechanisms, leading to different treatment response and clinical outcome. We know from gene expression and polymorphism studies that COPD is a polygenic disease involving multiple pathogenic signalling pathways.^10^, ^11^, ^12^ Whether and how the disease processes differ between the emphysema and bronchiolitis phenotypes of COPD remains to be investigated.

Large‐scale gene expression analysis using systems biology may help to address this question. Weighted gene coexpression network analysis (WGCNA) identifies correlations among genes across microarray samples, so it can detect clusters (modules) of highly correlated genes. It can also summarize such clusters using the module eigengene or an intramodular hub gene, it can relate modules to one another and to external clinical traits, and it can measure module membership.^13^ Building on WGCNA, the method known as differential coexpression (DiffCoEx) can identify changes in gene correlation patterns.^14^

Using differential gene expression analysis, Faner et al^15^ characterized genes differentially expressed between COPD patients with bronchiolitis or emphysema. They found that several B cell–related genes were up‐regulated in patients with emphysema but not in patients with bronchiolitis. We felt that simple up‐ and down‐regulation of differential gene expression were unlikely to capture the full complexity of COPD pathology, so we used WGCNA and DiffCoEx to re‐analyse the GSE69818 gene expression data set at the Gene Expression Omnibus database. Our aim was to begin to identify genes differentially coexpressed between the bronchiolitis and emphysema phenotypes of COPD as a way to develop promising biomarkers for diagnosis and as a way to identify molecular pathways involved in each phenotype.

## 2 MATERIALS AND METHODS

### 2.1 Affymetrix microarray data

Data from 50 gene expression profiles in the data set GSE69818, based on the Affymetrix Human Genome U219 Array, were downloaded from the Gene Expression Omnibus (www.ncbi.nlm.nih.gov/geo). This data set includes lung tissue samples from 70 COPD patients, including 38 patients of emphysema and 32 patients of bronchiolitis. The diagnosis of bronchiolitis and emphysema was based on the results of CT scan and lung function test, the bronchiolitis was defined as Diffusion Capacity for Carbon Monoxide of the Lung (DLCO) >80% Ref and absence of CT emphysema, and the emphysema was defined as DLCO < 80% Ref and/or CT emphysema, which was described in previous studies.^15^, ^16^, ^17^, ^18^ All patients were former smokers.

Data for the 49 386 probes representing 20 958 genes in this microarray were extracted using ENSEMBL Gene ID. When the expression level of a gene was measured using multiple probes, the expression level was calculated by averaging the level for all the probes. Gene expression was normalized using the robust multi‐array average algorithm implemented in the *limma* package in R software.^19^ Genes differentially expressed between the two COPD phenotypes were identified using the *RankProd* package in R. The difference in expression had to be associated with *P* < .05 in order to qualify for further analysis. In the end, 8150 differentially expressed genes (DEGs) were analysed further.

### 2.2 WGCNA

Comparability was assessed in terms of gene expression levels and connectivity. Connectivity was measured using the soft connectivity function in the WGCNA package, which drew on data from 5000 randomly selected genes. A WGCNA was constructed in the WGCNA package, and cluster analysis was performed using the flash clust function in the flash Clust package. Modules were identified using the cutreeHybrid function in the Dynamic Tree Cut package. Modules are groups of genes that have similar patterns of connection strengths with all other genes of the network and that usually share similar functions.^20^

The module eigengenes function in the WGCNA package was used to identify module eigengenes (MEs), and then, correlations between MEs and individual genes were assessed using signed module membership (MM). These correlations were used to determine whether a given gene belonged to a given module. Correlations between MEs and clinical traits were used to identify gene modules whose expression patterns were associated with particular clinical traits. Correlations between the expression level of a given gene and the presence of a clinical trait were defined as gene significance. The clinical traits involved in our study were two phenotypes of COPD (bronchiolitis and emphysema), Global Initiative for Chronic Obstructive Lung Disease (GOLD) grade from 1 ~ 4 and three levels of DLCO. The gene significance was calculated for all genes against each clinical trait in each data set and then displayed using the plot module significance routine in the WGCNA package.

To verify correspondence between modules and traits, cluster analysis of samples was performed based on gene expression level in each module and bar plot of MEs. For each module, gene connectivity was calculated as described above, and the genes in each module showing the highest connectivity were regarded as hub genes.

### 2.3 Differential coexpression analysis

The differential coexpression of genes between bronchiolitis and emphysema was analysed using the DiffCoEx algorithm, which operates differently from WGCNA. Briefly, unsigned Pearson correlation matrices called adjacency matrices were calculated separately for the microarray results from patients with the emphysema or bronchiolitis phenotype, and differences between these matrices were calculated. A topological overlap matrix was calculated from the matrix of correlation change. To find modules of genes differing in similar ways between the two phenotypes, genes were clustered by average hierarchical clustering, using the topological overlap matrix as a distance metric. Modules were defined using the ‘hybrid’ method of dynamic tree cutting. The tree was cut at a height of 0.93, requiring a minimum cluster size of 20 genes, and modules whose eigengenes branched at a height ≤0.2 were merged.

### 2.4 Gene function analysis and the protein‐protein interaction (PPI) network

Genes and hub genes in each module were functionally analysed using the clusterProfiler algorithm for gene ontology (GO) and pathway enrichment according to the Kyoto Encyclopedia of Genes and Genomes (KEGG).^21^ Interactions among genes were described using STRING^22^ and displayed using CytoScape.^23^

## 3 RESULTS

### 3.1 Identification of gene coexpression networks and modules

**Figure 1:**
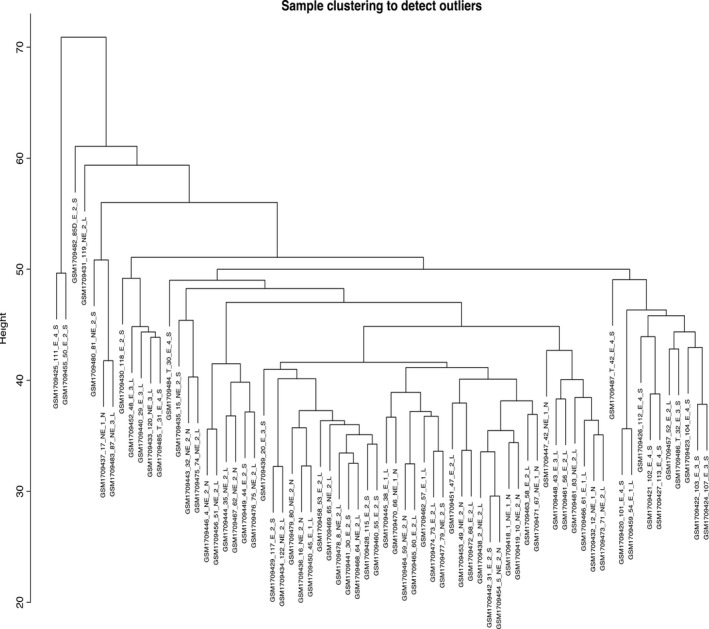
Hierarchical clustering of module hub genes that summarize the modules yielded in the clustering analysis. Branches of the dendrogram (the meta‐modules) represent together hub genes that are positively correlated

**Figure 2:**
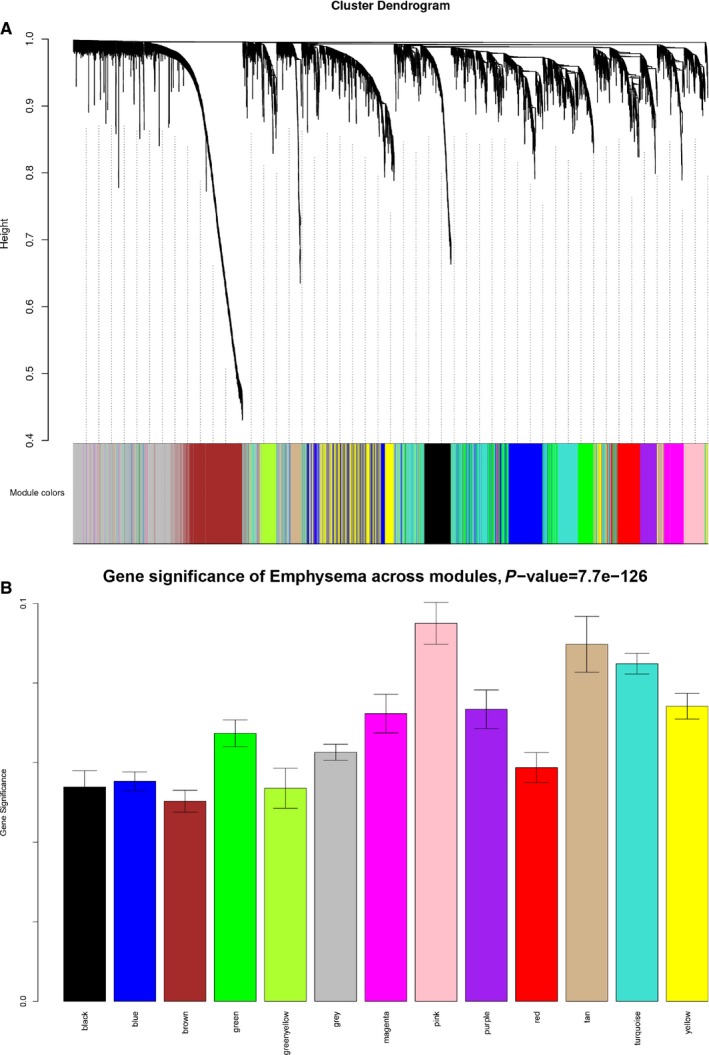
Identification of modules associated with the progression of COPD. A, Clustering dendrogram of genes, with dissimilarity based on topological overlap, together with assigned module colours. B, Distribution of average gene significance and errors in the modules associated with the progression of emphysema

Of the 70 patients with COPD, the clinical information of the samples involving GOLD and DLCO was summarized in Supplementary Table S1. A total of 8150 human genes were subjected to WGCNA, and genes exhibiting similar patterns of expression were grouped into modules via hierarchical average linkage clustering (Figure 1). Network topology was analysed using various soft threshold powers in order to ensure relatively balanced scale independence and mean connectivity. Power 4 was the lowest thresholding power for which the scale‐free topology fit index reached 0.85 (Supplementary Figure S1), so it was used to produce a hierarchical clustering tree (dendrogram) of the 8150 genes. This led to the identification of 12 modules (Figure 2), each of which had at least 50 genes; any module with fewer genes was merged with a similar module. In the end, the modules contained between 211 and 1772 genes. Modules were arbitrarily assigned colours: the smallest was green yellow, and the largest was grey. Grey genes were not divided into any modules.

### 3.2 Association of gene coexpression modules with clinical variables

WGCNA was used to correlate each module with all clinical traits available from NCBI. A *P* value was calculated for each module‐trait correlation. Of all the module‐trait correlations (Supplementary Figure S2), those of the greatest clinical interest or statistical significance were analysed further. For example, the tan module correlated positively with emphysema (cor = 0.28, *P* = .02), GOLD grade 4 (cor = 0.31, *P* = .009) and DLCO inferior to 60% (DLCO _S, cor = 0.25, *P* = .03). Genes in the tan module enriched in immunoregulation were consistent with those identified previously.^15^ The strongest association between module and trait was between the pink module and DLCO_S (cor = 0.48, *P* = 2E‐05; Supplementary Figure S2). Other significant correlations were found between the pink module and emphysema (cor = 0.34, *P* = .004) and GOLD grade 4 (cor = 0.33, *P* = .003). Therefore, the pink and tan modules were analysed further.

### 3.3 Functional enrichment and WGCNA clustering

**Figure 3:**
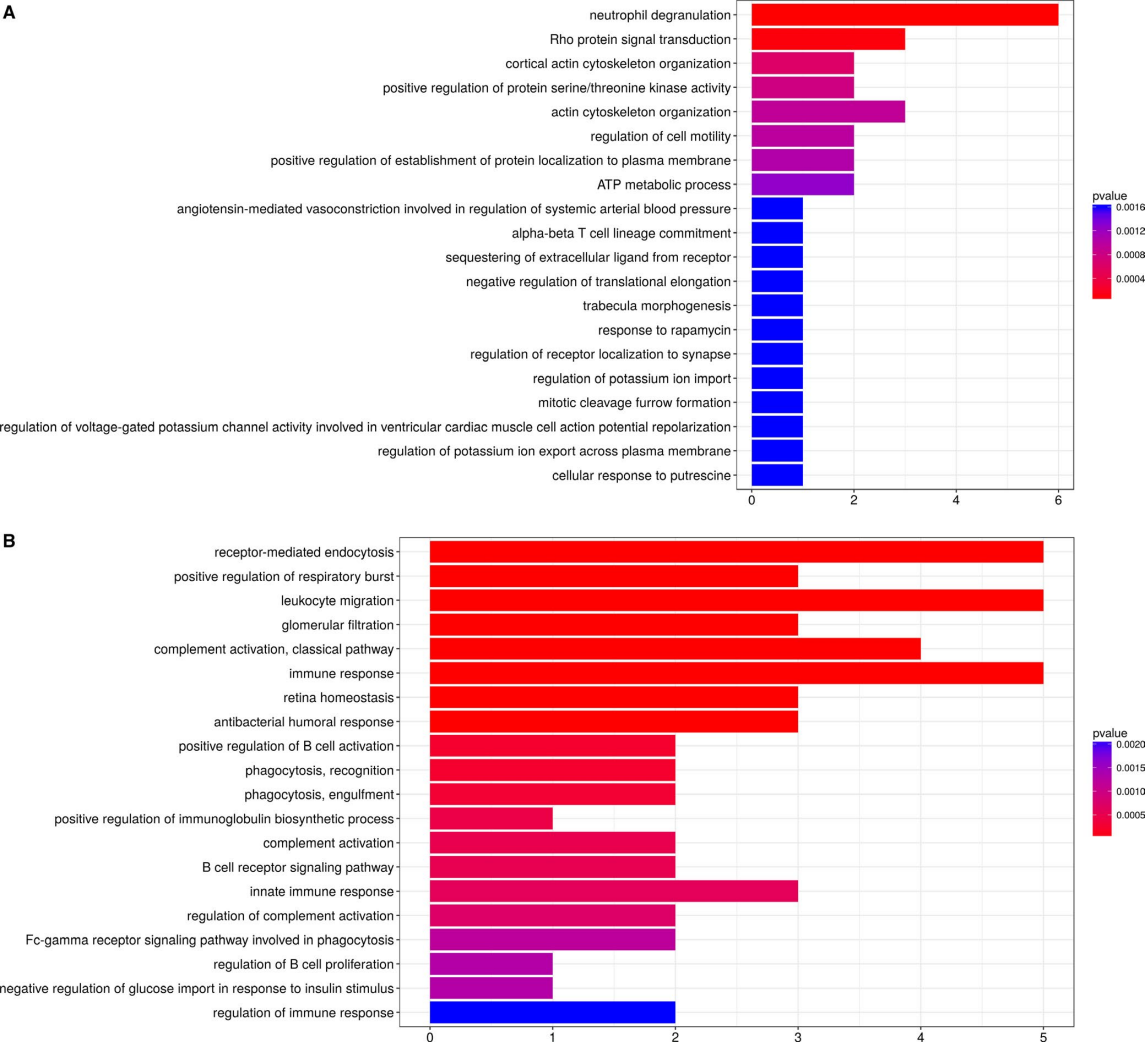
Gene ontology (GO) annotation of genes in the (A) pink and (B) tan module. The significant GO terms that conformed to *P* < .05 were screened. The *x*‐axis shows the number of entries enriched for specific GO‐BP: the longer the column, the greater the numbers of enriched entries. The *y*‐axis represents specific GO‐BP entries. Column colour is used to map the p value of specific functional items. Stronger red colour of the column indicates smaller *P* value of the corresponding enrichment, thus more significant enrichment

Given the similarity among genes within each module, the genes differentially expressed between the bronchiolitis and emphysema phenotypes in the pink and tan modules were functionally annotated and analysed for pathway enrichment. The top 20 GO and KEGG terms were extracted for further analysis, with a threshold of *P* < .05. Hub genes in the pink module (ROCK1, RHOG, RHOA, ATP6AP2, SNAP23 and PGRMC1) were enriched for the functions of neutrophil degranulation and Rho protein signal transduction (Figure 3A). Hub genes in the tan module (IGLV1‐40, JCHAIN, IGHA2, IGKV1D‐33, IGHA1, MZB1) were enriched mainly for immune‐related functions, such as immune response, B cell receptor signalling, regulation of B cell activation and proliferation (Figure 3B). Expression of CD79A, a component of the B cell receptor, is located at the centre of the coexpression network in the tan module (Supplementary Figure S3). Based on KEGG analysis, genes in the pink module were enriched mainly for the functions of oxidative phosphorylation, T cell receptor signalling, sphingolipid signalling, cAMP signalling and sphingolipid signalling (Supplementary Table S2).

### 3.4 Modules differentially coexpressed between emphysema and bronchiolitis phenotypes of COPD

**Figure 4:**
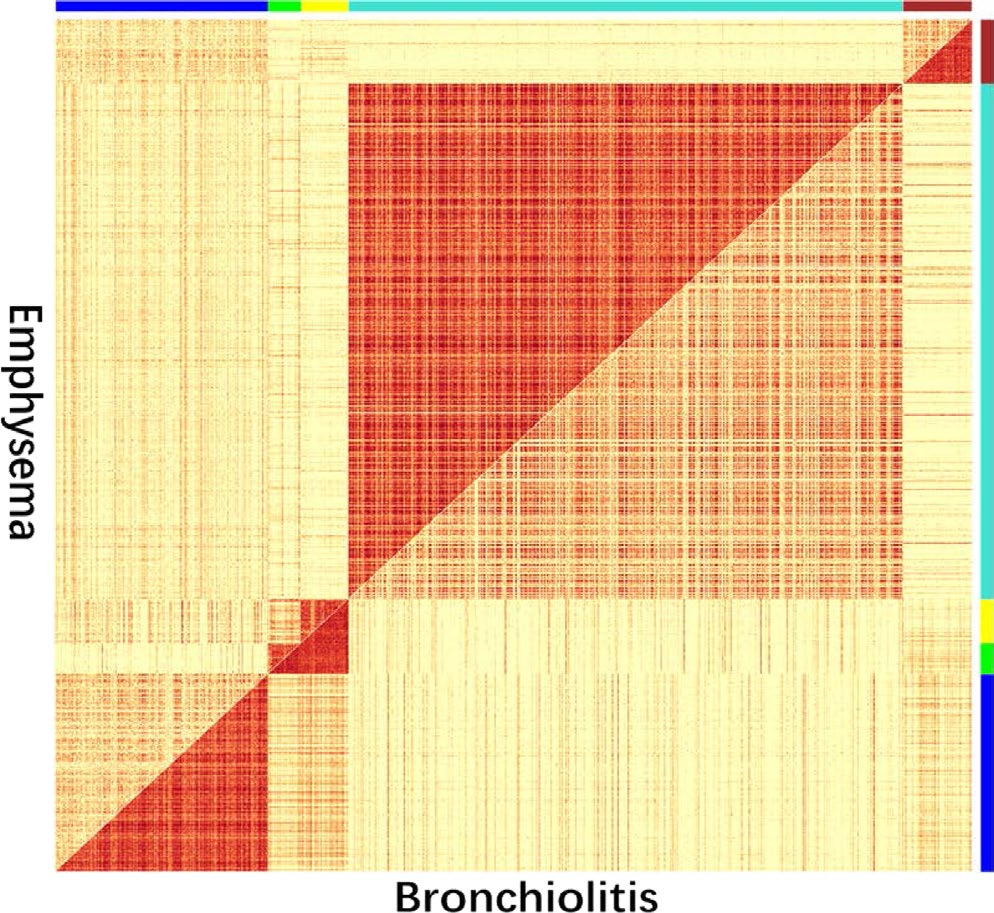
Differentially coexpressed modules between bronchiolitis and emphysema: comparative correlation heatmap. The upper diagonal of the main matrix shows a correlation between pairs of genes among the emphysema (red colour corresponds to higher correlations; yellow, lower correlations). The lower diagonal of the heatmap shows a correlation between the same gene pairs in bronchiolitis. Colour bars at the edge of the square show modules in which genes coexpressed differently between emphysema and bronchiolitis (second column); darker colours indicate higher mean expression levels

**Figure 5:**
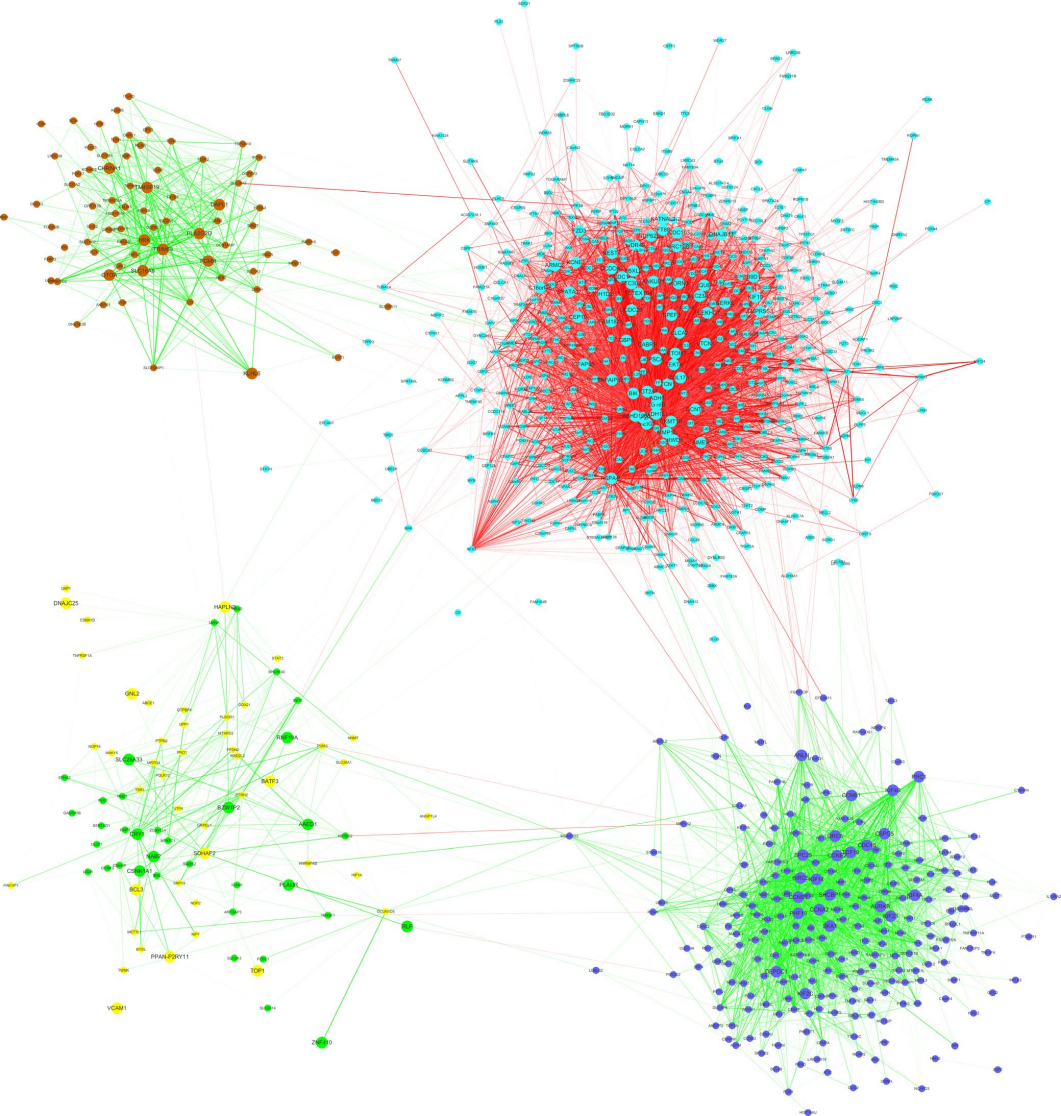
Differential coexpression network between emphysema and bronchiolitis. Dots in different colours represent genes in different modules. Edges shows different correlation between two genes; only Pearson δR differences （δR = |Remph|−|Rbronch|） reaching 0.7 are shown. Red lines show stronger correlation in emphysema than in bronchiolitis; green lines show stronger correlation in bronchiolitis

Of the 8150 genes analysed by DiffCoEx, 1001 were assigned to one of five modules, which were arbitrarily assigned colours (Figure 4). The blue and brown modules, containing 307 genes, were significantly more highly correlated with each other in patients with the bronchiolitis phenotype than in patients with the emphysema phenotype. The opposite was observed for the turquoise module, containing 606 genes. In this way, the modules showed differential coexpression patterns between emphysema and bronchiolitis. The differential coexpression network was also visualized using Cytoscape (Figure 5).

### 3.5 Functional and pathway enrichment analysis of DiffCoEx

The biological significance of the modules was assessed using gene set enrichment analysis with clusterProfiler.^21^ In the biological process category, the following four GO terms were enriched significantly in the turquoise module: cilium assembly, epithelial cilium movement involved in determination of left/right asymmetry, proteolysis and apoptotic mitochondrial changes (Table 1). Then, we identified IFT88, CCDC103, MMP10 and Bik as hub genes of the above four GO items, respectively. Moreover, FOXJ1 and RFX3, which interacted with many cilia‐related genes in our gene interaction network diagram (Supplementary Figure S4), magnify the induction of cilia‐associated genes.^24^ The blue module, in contrast, was enriched for genes involved in cell division, mitotic spindle midzone assembly, DNA replication initiation and G2/M transition of mitotic cell cycle (Table 1). The blue module was also significantly involved in three pathways (Supplementary Table S3): cell cycle, cellular senescence and p53 signalling. The brown module was strongly enriched for genes involved in signal transduction, such as pathways involved in the differentiation of CD4 and CD25‐positive α‐β regulatory T cells, response to interleukin‐1, and skeletal muscle tissue growth (Table 1). The genes of brown module were involved in the metabolism of fatty acids, such as alpha‐linolenic acid, glycerophospholipid and arachidonic acid (Supplementary Table S3). Hub genes in each module were listed in Table 2.

**Table 1:** GO enrichment analysis in differential coexpression modules

Module	Accession no.	Term	Count	*P*‐value	Gene
Turquoise	GO:0060271	Cilium assembly	5	2.93505E‐05	TEKT4,TTC30A,IQUB,IFT88,B9D1
	GO:0006493	Protein O‐linked glycosylation	2	.002436169	GCNT3,KCNE1
	GO:0002426	Immunoglobulin production in mucosal tissue	1	.002160627	GCNT3
	GO:0060294	Cilium movement involved in cell motility	1	.021403293	TEKT4
	GO:0 006 508	Proteolysis	3	.010494271	FBXL2,MMP10,TMPRSS3
	GO:0008637	Apoptotic mitochondrial changes	1	.0423613	Bik
Green	GO:0032436	Positive regulation of proteasomal ubiquitin‐dependent protein catabolic process	2	.000631427	CSNK1A1,RNF19A
	GO:0002082	Regulation of oxidative phosphorylation	1	.002220713	SLC25A33
	GO:2001268	Negative regulation of cysteine‐type endopeptidase activity involved in apoptotic signalling pathway	1	.002775206	PLAUR
Yellow	GO:0018293	Protein‐FAD linkage	1	.001234301	SDHAF2
	GO:0045082	Positive regulation of interleukin‐10 biosynthetic process	1	.001234301	BCL3
	GO:0042345	Regulation of NF‐kappaB import into nucleus	1	.002467231	BCL3
	GO:0045415	Negative regulation of interleukin‐8 biosynthetic process	1	.002467231	BCL3
Blue	GO:0051301	Cell division	9	2.02366E‐10	KIF2C,KIF14,SPC25,CCNA2,CCNB1,CCNE2,BIRC5,SKA1,CCNE1
	GO:0051256	Mitotic spindle midzone assembly	4	1.77904E‐10	KIF4B,KIF4A,KIF23,AURKB
	GO:0007018	Microtubule‐based movement	5	6.79539E‐08	KIF2C,KIF14,KIF4B,KIF4A,KIF23
	GO:0 006 270	DNA replication initiation	4	1.46023E‐07	CCNE2,ORC6,CCNE1,CDC45
	GO:0000281	Mitotic cytokinesis	4	1.0171E‐07	KIF4B,ANLN,KIF4A,KIF23
	GO:0000082	G1/S transition of mitotic cell cycle	4	9.55225E‐06	CCNE2,ORC6,CCNE1,CDC45
	GO:0000278	Mitotic cell cycle	4	9.93329E‐06	KIF2C,AURKB,BIRC5,SKA1
	GO:0000086	G2/M transition of mitotic cell cycle	3	.000727182	CCNA2,CCNB1,BIRC5
	GO:0008283	Cell proliferation	3	.011268622	KIF2C,TCF19,AURKB
Brown	GO:0002361	CD4‐positive, CD25‐positive, alpha‐beta regulatory T cell differentiation	1	.001665946	PLA2G2D
	GO:0048630	Skeletal muscle tissue growth	1	.001665946	CHRNA1
	GO:0070555	Response to interleukin‐1	1	.01764336	TRIM63

**Table 2:** Hub genes identified in both emphysema and bronchiolitis using differential coexpression analysis

Module	Hub genes
Turquoise	CCDC103, IFT88, BIK, DCDC2B, BEST4, TCTEX1D4, CLCA2, GBP6, VTCN1, SPATA17, TEKT4, TTC30A, CERKL, CFAP65, FBXL2, ALDH1L1, NME9, ANKUB1, WDR49, CEP19, UGT2A1, ADH6, HSPA4L, SLC23A1, GABRP, ARMC2, LRWD1, IQUB, NUP62CL, CCDC160, FZD3, PSCA, TCN1, LRRC10B, LRTOMT, DNAJB13, MMP10, PIH1D2, MEIG1, COL17A1, PLEKHG7, CCDC60, MORN3, ABHD12B, CKMT1B, GCNT3, ZG16B, C16orf46, B9D1, FAM187A, KIF19, KATNAL2, SPEF1, TNFAIP8L1, CYP2F1, KCNE1, TMPRSS3
Blue	KIF2C, DEPDC1, KIF14, SPC25, CSPG5, CCNA2, CCNB1, KIF4B, TCF19, ANLN, KIF4A, CCNE2, PHF19, KIF23, PRC1, SHCBP1, ORC6, AURKB, BIRC5, SKA1, CCNE1, CDC45
Brown	PLA2G2D, TRIM63, FCMR, DAPL1, CHRNA1, KLHL6, TM4SF19, HRK, OTOA, SLC16A6
Green	SLC25A33, RLF, BZW1P2, CSNK1A1, RNF19A, AAED1, NAB2, CRY1, ZNF410, PLAUR
Yellow	GNL2, VCAM1, BATF3, HRH1, DNAJC25, SDHAF2, HAPLN3, TOP1, PPAN‐P2RY11, BCL3

## 4 DISCUSSION

The present study identified coexpression of several B cell–related genes enriched in the tan module, which positively correlate with emphysema, which is consistent with the work of Faner et al^15^ and other groups that implicates antigen and immune processes in COPD pathogenesis.^25^, ^26^ Moreover, we also identified other vital genes using gene interaction network analysis (Supplementary Figure S3). For example, CD79A increases with increasing emphysema severity,^27^ and targeting the CD79A interacting genes CXCL13 and CXCR5 causes a decrease in the immune responses of allergic airway inflammatory process.^28^ This should be explored in greater detail in future studies.

In addition, we focused on differential coexpression rather than differential expression in an attempt to identify functional gene modules with different coexpression signatures in the two COPD phenotypes. Thus, we performed DiffCoEx analysis to identify transcriptome differences between the two phenotypes. In this approach, genes are clustered into modules according to how similarly their expression differs between the two phenotypes, and then, significant differences among these modules can be identified.

Ciliary function abnormalities have been associated with smoking and reduced mucociliary clearance in COPD,^29^ contributing to increased inflammation and infection.^30^ Motile multi‐ciliated cells, the dominant cell type in the airway epithelium, perform the critical function of transporting mucus and entrapped inhaled environmental contaminants up from the airways.^31^, ^32^ In the human airway epithelium, FOXJ1 is a key regulator of multi‐ciliated cell differentiation, inducing expression of cilia genes involved in the differentiation towards the multi‐ciliated cell lineage from basal progenitor cells. FOXJ1 expression in basal cells induces the expression of a panel of cilia‐associated genes, including CETN2, DNAH11, DNAI1, DNALI1, EFHC1, SPAG6, TEKT, TEKT2 and TUBA1A, and RFX3 functions as a transcriptional coactivator of FOXJ1.^24^ Eight of these genes (72%) were integrated into the large gene interaction network diagram of turquoise (Supplementary Figure S4). Prior work has shown that in the large airway epithelium, cigarette smoking in healthy individuals and COPD is associated with shorter cilia than healthy non‐smokers.^33^ A number of proteins related to IFT have been shown in model systems to directly affect cilia length.^34^ For instance, cells carrying homozygous IFT88 hypomorphic mutation fail to grow cilia with normal length.^35^ The results of our DiffCoEx identify IFT88 as a hub gene enriched in cilium assembly of the turquoise module, and we observed the interaction between IFT88 and FOXJ1 in the gene interaction network diagram in that module (Supplementary Figure S4). Previous research has also suggested that FOXJ1 overexpression reversed the CSE‐mediated inhibition of cilia growth.^36^ Whether and how these two genes work together to maintain the normal length of cilia remains to be further studied. Moreover, IFT88 is part of the intraflagellar transport machinery and helps maintain cilia motility.^37^, ^38^ Knockout of IFT88 or the other IFT‐B subunits IFT20 and IFT38 results in a ‘no cilia’ phenotype.^39^, ^40^ CCDC103, a hub gene enriched in epithelial cilium movement involved in determination of left/right asymmetry, is an oligomeric coiled‐coil protein that binds tightly to the ciliary axoneme and facilitates dynein attachment to it, thereby promoting ciliary motility. Patients with loss‐of‐function mutations in CCDC103 have largely static cilia.^41^ Our results suggest that future studies should clarify the pathogenesis of these cilium‐related genes and their potential value as therapeutic targets against the bronchiolitis phenotype.

Chronic mucus hypersecretion in COPD patients is associated with more frequent exacerbations, steeper lung function decline, more frequent hospitalization and higher mortality.^42^, ^43^, ^44^ Our analysis identified Bik as a hub gene in the turquoise module that is associated with apoptotic mitochondrial changes. Bik is anchored on the endoplasmic reticulum membrane, where it promotes calcium release from the endoplasmic reticulum, triggering mitochondrial apoptosis.^45^ Peptides derived from the Bik BH3 domain induce death of hyperplastic cells in bronchiolitis models, ultimately reducing the number of cigarette smoke‐induced mucous cells. However, most of the epithelial cells remain unharmed even when Bik is expressed.^46^ In fact, we failed to identify coexpressing hub genes related to normal cilium assembly and movement and promotion of mucous cell apoptosis in patients with the bronchiolitis phenotype of COPD, which may contribute to cough and expectoration in affected individuals.

Imbalance of proteases plays an important role in the development and progression of COPD. The MMP10 gene, which we identified here as a proteolysis related hub gene, is a potential COPD biomarker.^47^ It promotes emphysema development by influencing the proteolytic and inflammatory activities of macrophages.^48^ Knocking out the MMP10 gene in mice strongly attenuates emphysema‐like lung injury induced by cigarette smoke.^49^ Future study should examine MMP10 as a therapeutic target in emphysema.

Cigarette smoke may contribute to COPD pathogenesis by interfering with the removal of apoptotic cells (efferocytosis),^50^ and active RhoA inhibits efferocytosis by inducing the formation of stress fibres and focal adhesions as well as by inducing cell spreading.^51^ It has been reported that apoptotic cells are abundant in animal models of emphysema.^52^, ^53^ In addition, the RhoA/ROCK pathway may up‐regulate inflammatory genes via NF‐κB,^54^ and blockade of ROCK inhibits NF‐κB activation and production of proinflammatory cytokines.^55^ Our WGCNA suggests that Rho protein signal transduction may be related to the development of emphysema by regulating the release of inflammatory factors and reducing the clearance of apoptotic cells, thereby damaging the tissue. This hypothesis should be explored in future work.

The present study updates our perspective and highlights promising therapeutic targets, while the results of this study should be interpreted with caution in light of several limitations. First, normal lung tissues were not included in the study, potentially biasing the results to a certain extent. Second, we analyse the single platform of data set; the sample size is relatively small; clinical traits such as FEV_1_ and exacerbation history were absent in the original files; and we cannot perform a more comprehensive WGCNA analysis. Third, the results of WGCNA and DiffCoEx still need to be confirmed in another cohort. In addition, the differential gene expression reflects the outcome of biological processes during emphysema or bronchiolitis, or rather a consequence of the COPD disease itself is still unknown. Future work should pay attention to this issue.

## 5 CONCLUSIONS

This study showed that emphysema and bronchiolitis phenotypes of COPD are different in transcriptional level. Immune‐related processes, cilium assembly and movement, proteolysis, apoptotic mitochondrial changes, neutrophil degranulation and RhoA/ROCK signalling play a role in the pathological process of emphysema. Hub genes related to these biological processes may be biomarkers of emphysema. These findings should be verified and extended in future experimental work.

## CONFLICT OF INTERESTS

The authors declare that they have no conflict of interests.

## AUTHORS CONTRIBUTION

J Qin, T Yang, Y Shen and F Wen designed the study and drafted the manuscript. J Qin, T Yang, N Zeng, C Wan, L Gao, X Li and L Chen carried out the experiments, data collection and analysis. Y Shen and F Wen performed the data analysis and revised the manuscript. All the authors read and approved the final manuscript.

## ETHICS APPROVAL AND CONSENT TO PARTICIPATE

Not applicable.

## Data Availability

All data used to support the findings of the current study are available from Gene Expression Omnibus (https://www.ncbi.nlm.nih.gov/geo/query/acc.cgi?acc=GSE69818).
