# Supplementary material for: Differential coexpression networks in bronchiolitis and emphysema phenotypes reveal heterogeneous mechanisms of chronic obstructive pulmonary disease
Source: J Cell Mol Med. 2019 Aug 16;23(10):6989–99. doi: 10.1111/jcmm.14585 (PMC6787516; doi:10.1111/jcmm.14585)
Supplement: Supplementary file 1 [file JCMM-23-6989-s001.docx]

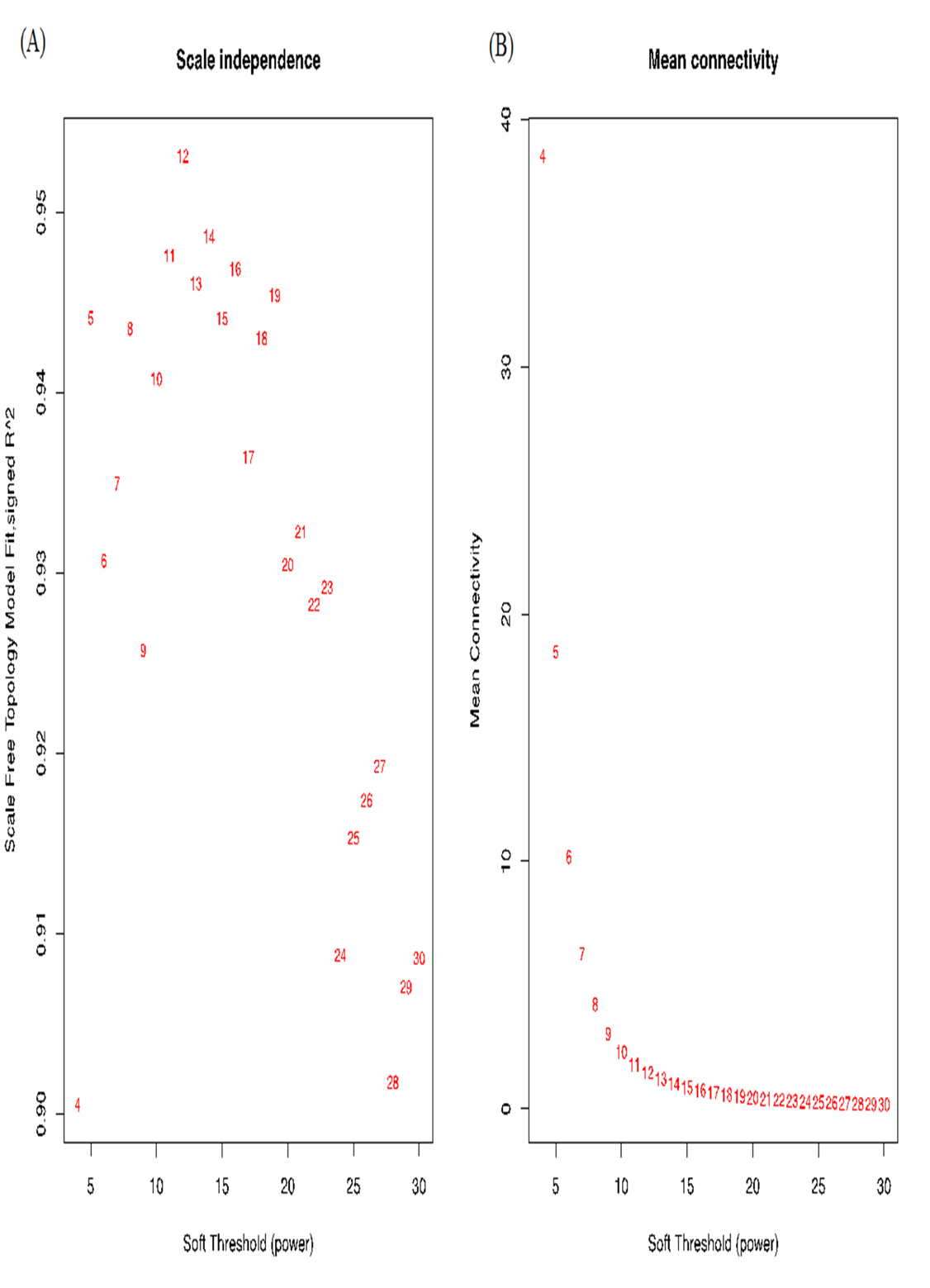


Supplementary Figure 1. Determination of soft‐thresholding power in the weighted gene co‐expression network analysis (WGCNA). (A) Analysis of the scale‐free fit index for various soft‐thresholding powers (β). (B) Analysis of the mean connectivity for various soft‐thresholding powers.


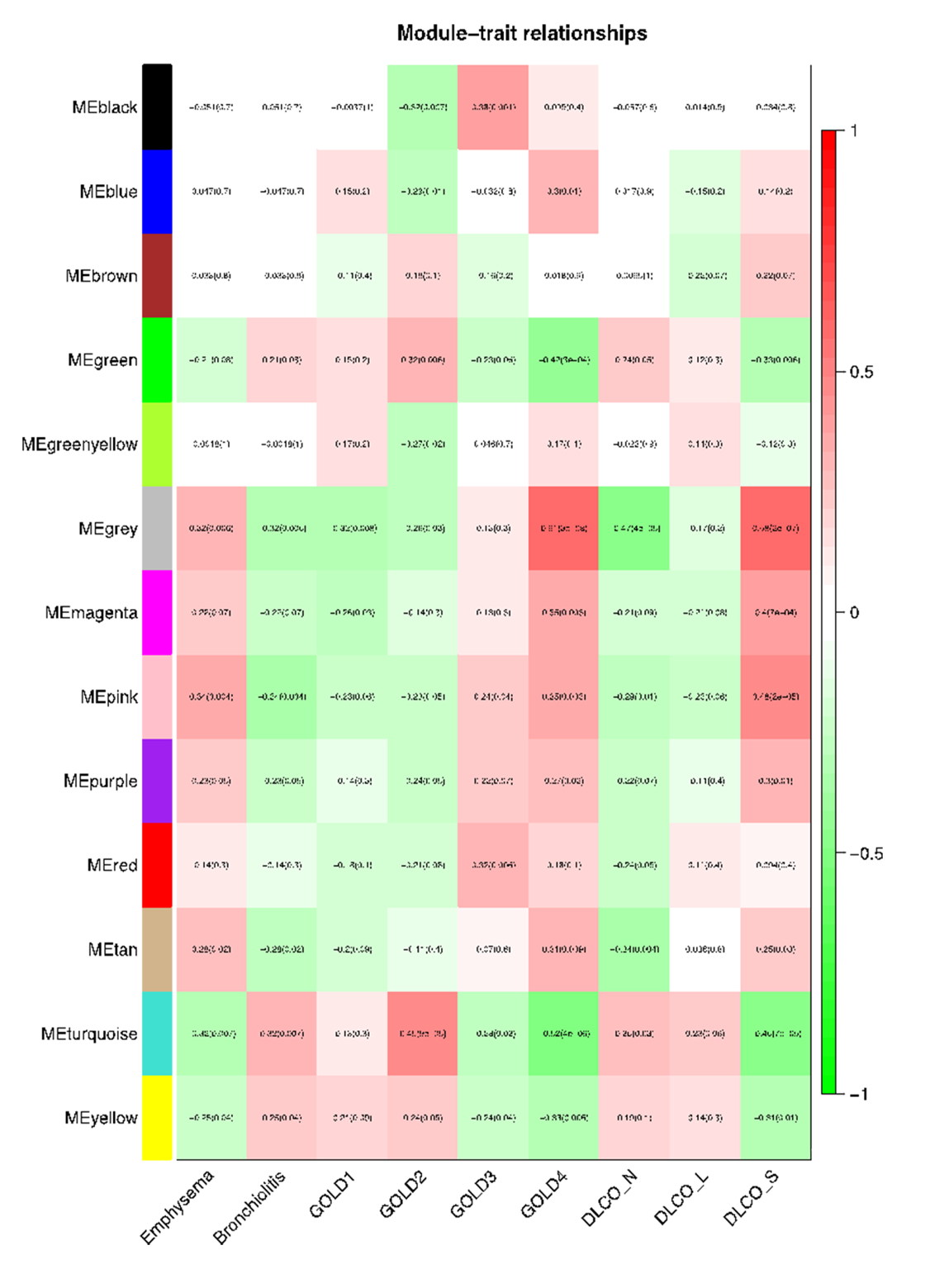
Supplementary Figure 2. Module-feature associations: Each row corresponds to a module Eigengene and each column to a clinical feature. Each cell contains the corresponding correlation in the first line and the P-value in the second line. The table is color-coded by correlation according to the color legend. DLCO_N refers to DLCO > 80%; DLCO_L, DLCO 60-80%; and DLCO_S, DLCO < 60%. Positive correlations are red; negative correlations, blue.


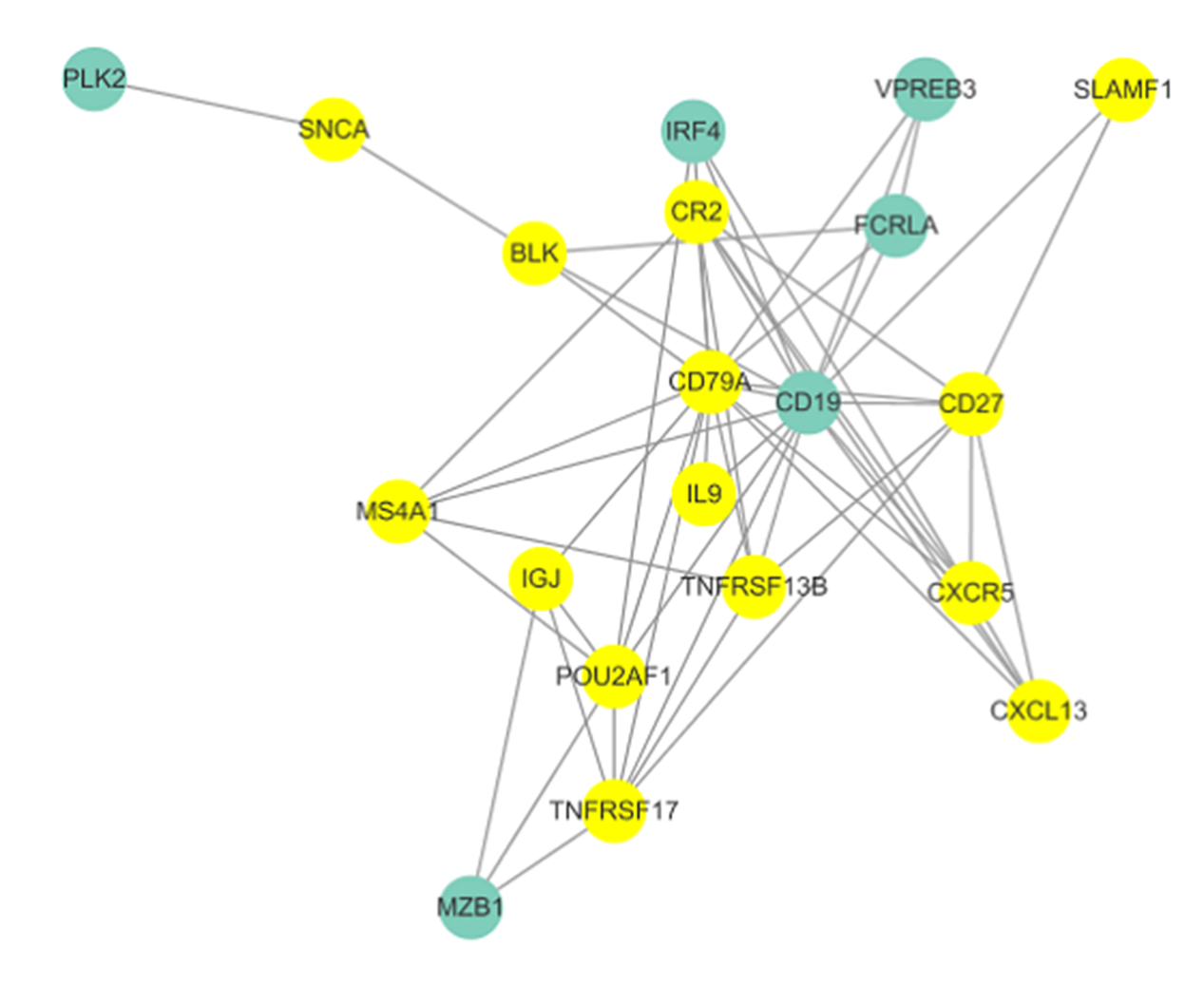


Supplementary Figure 3. Gene interaction network in the tan module. Connections between genes represent interaction between those genes. Yellow nodes represent genes of GO:0002376 ("immune system process") that appear in the primary literature.


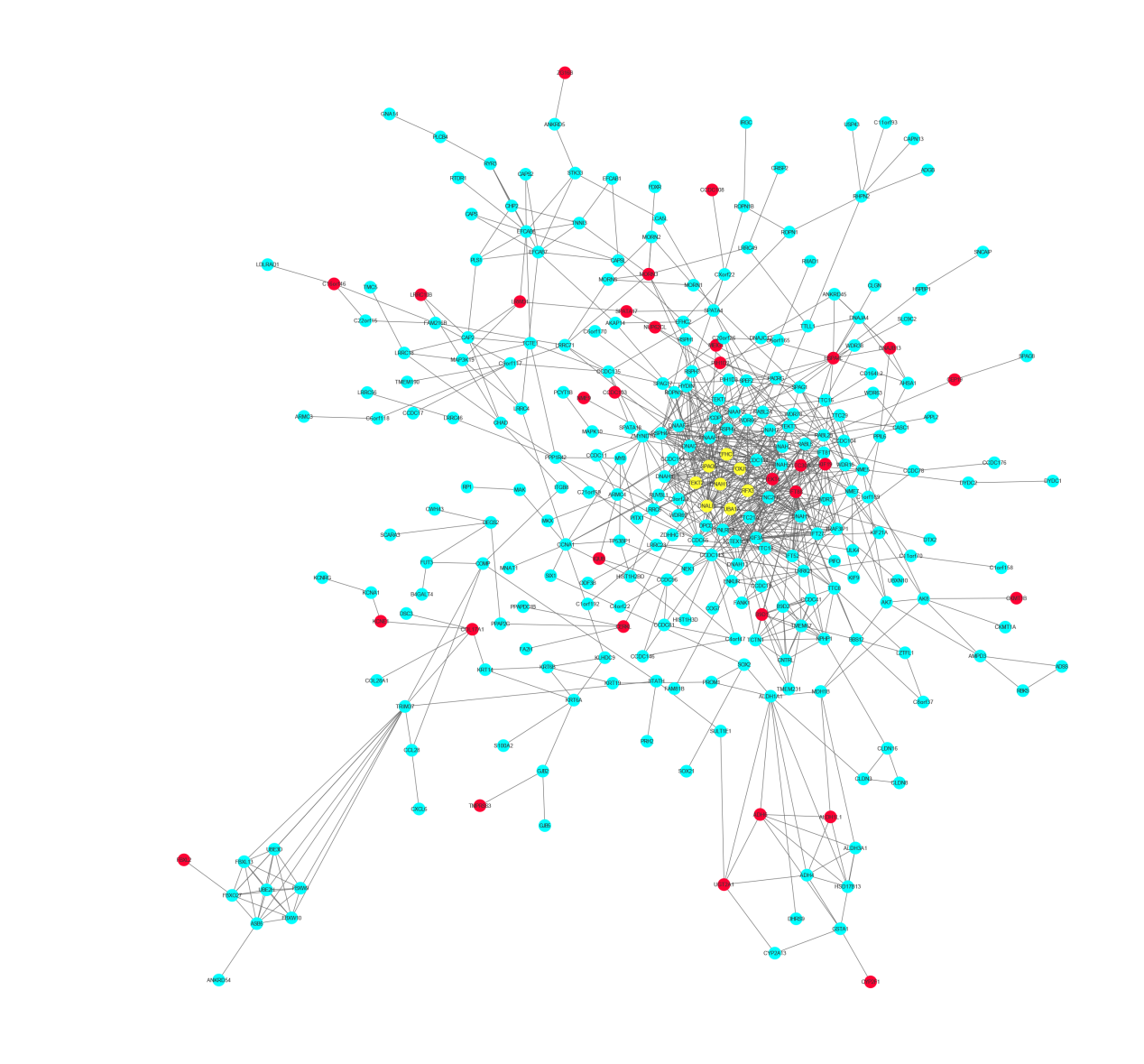
Supplementary Figure 4. Gene interaction network in the turquoise module: 604 genes were classified in the turquoise module in patients with emphysema. Connections between genes represent interactions between those genes. Red nodes represent hub genes. The yellow nodes indicates FOXJ1, its co-activator and a panel of known cilia-associated genes induced by FOXJ1 (RFX3, FOXJ1, TUBA1A, TEKT2, EFHC1, DNAH11, DNALI1 and SPAG6), which occupy a central position in the main network.
