# Supplementary material for: Differential coexpression networks in bronchiolitis and emphysema phenotypes reveal heterogeneous mechanisms of chronic obstructive pulmonary disease
Source: J Cell Mol Med. 2019 Aug 16;23(10):6989–99. doi: 10.1111/jcmm.14585 (PMC6787516; doi:10.1111/jcmm.14585)
Supplement: Supplementary file 2 [file JCMM-23-6989-s002.docx]

**Supplementary Table 1：Characteristics of COPD patients with bronchiolitis and emphysema.**

| Clinical traits | Emphysema | Bronchiolitis |
| --- | --- | --- |
| GOLD 1 | 5 | 6 |
| GOLD 2 | 17 | 24 |
| GOLD 3 | 7 | 2 |
| GOLD 4 | 9 | 0 |
| DLCO<60%（S） | 22 | 3 |
| DLCO 60%~80%（L） | 16 | 14 |
| DLCO>80%（N） | 0 | 15 |

**Supplementary Table 2：KEGG Pathways in Coexpression Modules.**

| Module | Accession no. | Term | Count | P-value | Gene |  |
| --- | --- | --- | --- | --- | --- | --- |
| Pink | hsa00190 | Oxidative phosphorylation | 4 | 0.000161937 | ATP5A1, SDHD, NDUFA5, NDUFS1 | |
|  | hsa04660 | T cell receptor signaling pathway | 3 | 0.00124617 | MAP2K2, DLG1, RHOA | |
|  | hsa04270 | Vascular smooth muscle contraction | 3 | 0.001812504 | MAP2K2, ROCK1, RHOA | |
|  | hsa04071 | Sphingolipid signaling pathway | 3 | 0.00185817 | MAP2K2, ROCK1, RHOA | |
|  | hsa04611 | Platelet activation | 3 | 0.00195166 | ROCK1, SNAP23, RHOA | |
|  | hsa04022 | cGMP-PKG signaling pathway | 3 | 0.00423843 | MAP2K2, ROCK1, RHOA | |
|  | hsa04024 | cAMP signaling pathway | 3 | 0.007811693 | MAP2K2, ROCK1, RHOA |  |

**Supplementary Table 3：KEGG Pathways in Differential Coexpression Modules.**

| Module | Accession no. | | Term | | Count | P-value | Gene |
| --- | --- | --- | --- | --- | --- | --- | --- |
| Turquoise | hsa00980 | Metabolism of xenobiotics by cytochrome P450 | | 2 | | 0.007063865 | ADH6, CYP2F1 |
|  | hsa00670 | One carbon pool by folate | | 1 | | 0.033670703 | ALDH1L1 |
|  | hsa00512 | Mucin type O-glycan biosynthesis | | 1 | | 0.05553306 | GCNT3 |
| Green | hsa04710 | Circadian rhythm | | 1 | | 0.012654395 | CRY1 |
|  | hsa04340 | Hedgehog signaling pathway | | 1 | | 0.020022615 | CSNK1A1 |
|  | hsa04610 | Complement and coagulation cascades | | 1 | | 0.030362267 | PLAUR |
| Yellow | hsa04668 | TNF signaling pathway | | 2 | | 0.001422265 | VCAM1, BCL3 |
|  | hsa03008 | Ribosome biogenesis in eukaryotes | | 1 | | 0.043674644 | GNL2 |
| Blue | hsa04110 | Cell cycle | | 6 | | 7.70E-10 | CCNA2, CCNB1, CCNE2, ORC6, CCNE1, CDC45 |
|  | hsa04218 | Cellular senescence | | 4 | | 1.52505E-05 | CCNA2, CCNB1, CCNE2, CCNE1 |
|  | hsa04115 | p53 signaling pathway | | 3 | | 4.84325E-05 | CCNB1, CCNE2, CCNE1 |
| Brown | hsa00592 | alpha-Linolenic acid metabolism | | 1 | | 0.010045055 | PLA2G2D |
|  | hsa00591 | Linoleic acid metabolism | | 1 | | 0.011350299 | PLA2G2D |
|  | hsa04975 | Fat digestion and absorption | | 1 | | 0.015692824 | PLA2G2D |
|  | hsa00565 | Ether lipid metabolism | | 1 | | 0.018725014 | PLA2G2D |
|  | hsa00590 | Arachidonic acid metabolism | | 1 | | 0.025632335 | PLA2G2D |
|  | hsa00564 | Glycerophospholipid metabolism | | 1 | | 0.040628934 | PLA2G2D |
